# The Efficacy and Safety of Deferasirox Monotherapy as a Second-Line Treatment in Transfusion-Dependent Thalassemia with Iron Overload

**DOI:** 10.3390/jcm14176212

**Published:** 2025-09-03

**Authors:** Manatchaya Pongamnuaykrit, Adisak Tantiworawit, Piangrawee Niprapan, Teerachat Punnachet, Nonthakorn Hantrakun, Pokpong Piriyakhuntorn, Thanawat Rattanathammethee, Sasinee Hantrakool, Chatree Chai-Adisaksopha, Ekarat Rattarittamrong, Lalita Norasetthada, Pimlak Charoenkwan

**Affiliations:** 1 Division of Hematology, Department of Internal Medicine, Faculty of Medicine, Chiang Mai University, Chiang Mai 50200, Thailand; minkmanat@gmail.com (M.P.);; 2 Division of Hematology and Oncology, Department of Pediatrics, Faculty of Medicine, Chiang Mai University, Chiang Mai 50200, Thailand

**Keywords:** deferasirox, transfusion-dependent thalassemia, thalassemia

## Abstract

Deferasirox (DFX) is an oral iron chelator for thalassemia patients with iron overload. DFX was FDA-approved as a first-line treatment for chronic iron overload. In Thailand, DFX was indicated as second-line therapy for patients unresponsive to deferiprone. **Objectives**: This study aimed to investigate the efficacy and safety of DFX monotherapy. **Methods**: All transfusion-dependent thalassemia patients who received second-line DFX monotherapy were identified from the thalassemia registry between May 2007 and May 2022. The primary endpoint was the change in body iron stores, measured by serum ferritin at week 24. At treatment end, patients with a serum ferritin (SF) level < 1000 ng/mL in transfusion-dependent thalassemia (TDT) were categorized as the ferritin response group. Multivariate analysis identified factors driving group differences. **Results**: Forty-two patients were enrolled with a mean age of 35.5 (13–57) years. Of these, 73.81% had beta-thalassemia. The median initial DFX dose was 20.26 (17.85–22.22) mg/kg/day, with a median treatment follow-up of 2 (1.80–2.45) years. Median SF was decreased from 2516 (1712 to 3065) ng/mL to 1027.5 (598–1867) ng/mL (*p* < 0.001). Of 21 (50%) patients in the ferritin response group, independent factors were age > 15 years and lower initial SF, with OR = 7.13 (95% CI 1.05–48.49, *p* = 0.045) and OR = 0.93 (95% CI 0.87–1.00, *p* = 0.039). The most common adverse events were gastric irritation symptoms (11.90%). **Conclusions**: Deferasirox is an effective oral iron chelator for thalassemia, with manageable side effects. Half of patients reached target SF levels. Adults (>15 years) with lower initial SF levels had a better response to DFX.

## 1. Introduction

Thalassemia is the most common genetic hematologic disorders in Thailand. About 15–20% of the thalassemia patients have severe degrees of anemia and require regular packed red blood cell transfusions to maintain hemoglobin levels between 9 and 10 g/dL [1]. Adequate blood transfusion can prevent extramedullary erythropoiesis [2]. Although transfusions improve survival, they inevitably lead to iron overload, as each packed red blood cell unit contains 200–250 mg of iron which is around 100–150 times the normal iron absorption [3]. Iron overload is diagnosed by serum ferritin levels above 1000 ng/mL or liver iron concentration (LIC) above 7 mg Fe/g dry weight [4]. Iron overload is harmful to many organs, leading to complications such as liver cirrhosis, heart failure, growth retardation, and various endocrine disorders [4].

The management of iron overload is iron chelating therapy. Currently, three iron chelating agents, administered through parenteral and oral formulations, are employed in the management of iron overload. Deferoxamine (DFO), the most studied chelator, is effective but requires parenteral administration, leading to poor compliance [5,6]. Deferiprone (DFP), the first oral chelator, improves compliance and may be more effective when combined with DFO, especially for reducing cardiac iron [7]. Deferasirox (DFX), another oral chelator, offers once-daily dosing with better compliance [8,9]. The starting dose is 20 mg/kg/day [10,11]. A dose of 30 mg/kg/day is usually required to achieve a negative iron balance [11]. Previous studies show it significantly reduces LIC and myocardial iron with a favorable safety profile [12]. One study reported that doses of DFX greater than 30 mg/kg/day effectively reduced iron burden with safety and no concern regarding renal or liver function [13]. Common side effects of DFX include gastrointestinal symptoms, rash and reversible elevations in alanine transaminase and serum creatinine [14,15,16]. Although renal toxicity is a rare side effect of DFX, there have been reports of acute interstitial nephritis or Fanconi syndrome due to this medication [17,18,19].

DFX received approval from the US FDA and was also recommended by The Thalassemia International Federation (TIF) for the first-line treatment of chronic iron overload due to blood transfusions in adult and pediatric patients (aged two years and over). In Thailand, DFX was included in Thailand’s National List of Essential Medicines (NLEM) in 2017. However, it was only approved as a first-line treatment for patients aged two to six years old. The efficacy and safety have been reported previously [20]. However, for patients aged more than six years, DFX was indicated as the second-line therapy for patients who were unresponsive to DFP or exhibiting adverse events to DFP. As the previous data showed, a total of 58 (55.24%) patients receiving DFP monotherapy were in the achievable group, defined as serum ferritin <1000 ng/mL in transfusion-dependent thalassemia (TDT) and <800 ng/mL in non–transfusion-dependent thalassemia (NTDT) for two consecutive visits [21]. This means that nearly 50% of patients had inadequate control of iron overload and required deferasirox as second-line therapy. There was limited data on the efficacy of DFX as a second-line treatment. To date, there is limited published data have examined the real-world efficacy and safety of DFX monotherapy as a second-line agent following DFP discontinuation in Southeast Asian populations. Therefore, this study aims to evaluate the efficacy and safety of DFX monotherapy in transfusion-dependent thalassemia (TDT) patients receiving it as second-line treatment in real-world clinical practice.

## 2. Materials and Methods

### 2.1. Target Patients and Study Design

This study was conducted at Chiang Mai University Hospital, Chiang Mai, Thailand. The data were retrieved from a thalassemia web-based database registry. All thalassemia patients aged more than six years or older who received DFX monotherapy as a second-line therapy were identified from our thalassemia database between May 2007 and May 2022. Inclusion criteria were TDT patients, the diagnosis and type of thalassemia were confirmed by high-performance liquid chromatography (HPLC). Inadequate response to first-line iron chelation treatment is defined as a serum ferritin level greater than 2500 ng/mL after one year of prior iron chelator therapy, or a decrease in serum ferritin of less than 15% after two years of treatment. Exclusion criteria were patients with underlying liver disease (elevated AST or ALT level three times or higher than the normal range), renal parenchymal disease (estimated glomerular filtration rate (eGFR) by Cockcroft-Gault equation less than 60 mL/min/1.73 m^2^), cardiovascular disease, other coexisting hemolytic anemias, any cancer, and chronic infection [21]. DFX monotherapy was administered as a dispersible tablet at a dose of 25–40 mg/kg/day.

Medical records that included sex, age, thalassemia type, splenectomy status, other underlying diseases, previous iron chelation, and initial SF were collected before starting DFX therapy. Dose of DFX, SF, pretransfusion hemoglobin (Hb), adverse event, and laboratory, including serum creatinine, AST (aspartate aminotransferase), and ALT (alanine aminotransferase) were collected every three months. Data collection continued for 24 months since the initiation of DFX.

The study protocol was reviewed and approved by the Ethics Committee of the Faculty of Medicine, Chiang Mai University, Chiang Mai, Thailand (approval code: MED-2565-08997). All procedures were conducted in accordance with the principles outlined in the Declaration of Helsinki. The study did not receive external funding support, and the requirement for informed consent was waived by the committee.

### 2.2. Efficacy Assessments

Serum ferritin levels were measured at baseline, every three months, and at the conclusion of the study. Following treatment, patients were categorized into two groups: the ferritin response group, defined as achieving a target SF level of <1000 ng/mL at the end of the study, and the non-ferritin response group, defined as failure to achieve the target SF level by study completion. Demographic and clinical characteristics were compared between the two groups to identify factors associated with treatment response.

### 2.3. Adverse Event Assessments

The adverse events of DFX were gastrointestinal irritation such as nausea and vomiting, abdominal pain, and diarrhea, skin rash, liver transaminitis, and elevation in serum creatinine. These data were collected from medical records. Patients who discontinue DFX due to adverse effects or are lost to follow-up before the end of the two years or death occurs will be considered dropouts from the study.

### 2.4. Statistical Analyses

The calculation of the sample size for a cohort study is analyzed by a two-sample paired-means test. In a previous study of the safety and efficacy of DFX in patients with TDT, the median (range) of baseline SF was reported to be 1254 (276–145,000) ng/mL [22]. Treatment of DFX was continued for four years. At the end of the study, SF was reported to be 1124 (365–5598) ng/mL. We estimate the mean and SD from the median (range) as Hozo et al. [23]. Mean (SD) of baseline serum ferritin was 4321 (2370.67) ng/mL and the end of the study was 2052.75 (872.17) g/mL. This study shows that the SF level at the end of the study will be different from the baseline value by at least 20% = 864.20. If the correlation is 0.5–0.6 with a confidence level of 95%. The sample size required for achieving an 80% power of test. The estimated sample size was 43 patients.

The Statistics Package of the Stata Statistical Software: Release 17. (College Station, TX, USA: StataCorp LLC) was used for statistical analysis. Categorical variables were described with counts and percentages and continuous variables were described with mean ± SD or median with interquartile range (IQR) or range as appropriate context. Transforming data was used to improving normality of a variable. Comparisons between groups were performed using pair/unpair *t*-test and signed-rank/the Mann–Whitney U test for continuous variables, and the chi-square test for categorical variables. For repeatedly measured continuous outcomes, the Repeated Measures Mixed Model was used. Associations were assessed by calculating odds ratios (ORs) with 95% confidence intervals (CIs). Variables with a *p*-value < 0.10 in the univariate analysis were entered into the multivariate model. Multiple logistic regression analysis was conducted to identify factors independently associated with group differences. Statistical significance was defined as *p* < 0.05.

## 3. Results

### 3.1. Baseline Characteristics

Forty-two patients were enrolled in the study. The subjects were predominantly male (52.38%) with a median age of 35.5 (range 13–57) years. Of these, 73.81% were beta-thalassemia and 28.57% were splenectomy. The median initial DFX dose was 20.26 (range 17.85–22.22) mg/kg/day and the median follow-up duration of treatment was two (range 1.80–2.45) years. During treatment, the median maximum dose of DFX was 27.19 (range 21.73–32.37) mg/kg/day and 15 patients (35.71%) were treated with a dose of >30 mg/kg/day. The median initial SF level was 2516 (range 1712–3065) ng/dL and the mean pre-transfusion Hb was 8.1 ± 1.60 g/dL. The previous iron chelators were DFO monotherapy (33.33%), DFP monotherapy (64.29%), and combination therapy of DFO and DFP (2.38%). The median transfusion was 0.75 (0.5–1) units per month. Baseline characteristics are shown in Table 1.

### 3.2. Efficacy of Deferasirox

**Figure 1:**
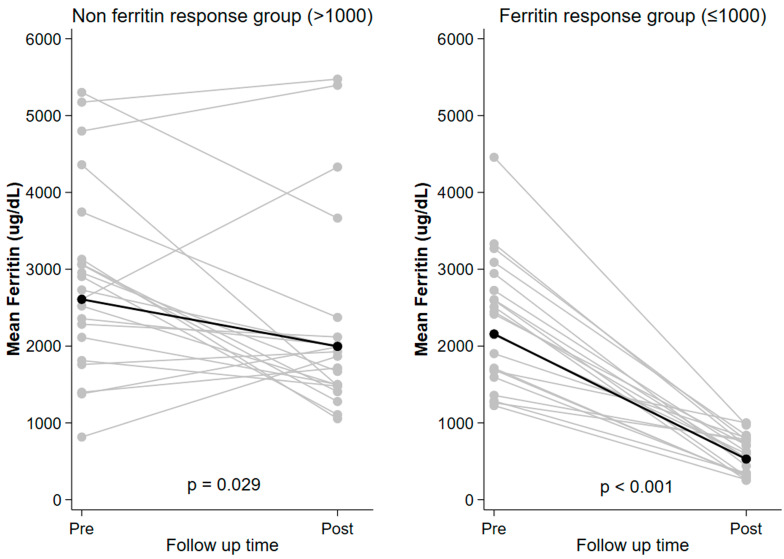
Baseline and Endpoint Serum Ferritin Levels Across Groups.

The initial SF level and last SF level after DFX treatment are shown in Figure 1 compared between the ferritin response group and the non-ferritin response group.

The mean (95% CI) SF level in the ferritin response group was changed from 2157.2 (1827.1–2547.0) ng/mL to 528.9 (428.8–652.1) (*p* < 0.001). In the non-ferritin response group, the mean (95% CI) SF was changed from 2608.6 (95% CI 2109.1–3226.3) to 1995.7 (95% CI 1608.6–2476.1) (*p* = 0.029). Notably, the ferritin response group demonstrated a 75.5% reduction, which was approximately three times greater than the 23.5% reduction observed in the non-ferritin response group. While baseline ferritin levels did not differ significantly between the two groups (*p* = 0.165), a significant difference was observed in the final ferritin levels at the end of the study (*p* < 0.001).

At a median follow-up of two (1.80–2.45) years, the median SF decreased from 2516 (1712 to 3065) to 1027.5 (598–1867) ng/mL. (*p* < 0.001). Out of 42 patients, 21 (50.0%) were in the ferritin response group and 21 patients (50.0%) were in the non-ferritin response group. From univariate analysis, the significant factors associated with the ferritin response group were age (OR = 8.64; 95% CI, 1.56–47.78; *p* = 0.013), thalassemia type (OR = 7.12; 95% CI, 1.28–39.58; *p* = 0.025), and initial serum ferritin (OR = 0.99; 95% CI, 0.99–1.00; *p* = 0.083). Between the two groups, the significant factors associated with the ferritin response group according to the result of the multivariate analysis were adult (age > 15 years) and initial lower SF with OR = 7.13 (95% CI 1.05 to 48.49, *p* = 0.045) and OR = 0.93 (95% CI 0.87 to 1.00, *p* = 0.039) as shown in Table 2. Adjusted by age group and thalassemia type, for every 100 ng/mL increase in initial serum ferritin levels, there is a 1.2% decrease in the predictive probability of ferritin response group (95% CI −2.18 to −0.21, *p* = 0.017).

### 3.3. Adverse Events

Of the 42 patients, one patient (2.38%) had severe drug-related adverse events. The most common adverse events were gastric irritation symptoms (11.90%). The side effect was nausea in four patients (9.52%) followed by vomiting that was documented in one patient (2.38%). Skin rash was not found in this study. Elevation of aminotransferase enzyme (more than two times of baseline) was found in two cases. Seven patients (16.67%) had transiently increased serum creatinine (increase > 33% baseline). None of the adverse events led to death during the study period. One patient experience severe vomiting and cannot continue taking DFX. This patient was considered a dropout from the study at three months.

## 4. Discussion

Our study demonstrated the long-term efficacy of DFX monotherapy as a second-line treatment in patients with TDT. A significant reduction in SF levels was observed, with half of the cohort achieving the target SF threshold. Independent factors associated with achieving ferritin response included being an adult (age > 15 years) and having a lower baseline SF level.

The findings of our study regarding the ferritin response group align with the results of a previous study on DFX monotherapy conducted in 237 patients with beta-thalassemia and transfusional iron overload [12]. In this previous study, it was observed that the median serum SF decreased by 341 ng/mL over the 52 weeks, with a range from −9193 to 2835 ng/mL. Remarkably, our study also observed a more substantial reduction in the median SF in the adult population compared to the pediatric population, mirroring the findings of this previous research [12]. A recent study on a dispersible tablet of GPO-DFX monotherapy among children with TDT showed that the median of baseline SF 2383 (1314–3192) ng/mL decreased to 1478 (888–2858), 1038 (427–2672) and 1268 (1077–3101) ng/mL at the end of first, second and third years, respectively [20]. It appears to demonstrate a decrease in median SF the same as our study that was the median SF of the pediatrics group (age ≤ 15) changes from 2480.5 (1936.5–2842.5) to 1821 (1278–2066) ng/mL in two-year treatment of DFX. However, in those studies, ferritin levels were reduced even more compared to our study. This might be explained by the difference in the study population as our study enrolled older children with second-line treatment and a shorter duration of follow-up. These findings underscore the significance of adjusting DFX doses promptly in response to serum ferritin levels to ensure patients attain their therapeutic objective of maintaining or reducing their iron burden. It was observed that dose escalations to 25 mg/kg/day were necessary for 59% of patients who had not reached their target serum ferritin levels. This highlights the importance of vigilant monitoring and individualized dose adjustments to optimize treatment outcomes in patients receiving DFX therapy.

We observed that being an adult (age > 15 years) was associated with a better response to DFX treatment. Several factors may explain the observation that adults demonstrate a better response to deferasirox (DFX) compared with children. Pharmacokinetic differences, particularly faster hepatic metabolism and clearance in children, may reduce systemic exposure to the drug at equivalent weight-based doses [24]. In addition, adults often exhibit higher treatment adherence due to greater disease awareness, whereas adherence in pediatric patients may be influenced by caregiver–child dynamics [25]. Finally, fluctuations in weight during growth spurts may result in inadvertent underdosing in children, whereas adult body weights are more stable, allowing more consistent dosing. Previous studies have demonstrated that deferasirox (DFX) monotherapy is effective in reducing serum ferritin levels in patients with beta-thalassemia and transfusional iron overload [12]. In our study, alpha-thalassemia was significantly associated with the ferritin response group in the univariable analysis, but this association was not maintained in the multivariable analysis. This observation may be attributed to the fact that most patients in the alpha-thalassemia group had HbH disease, which is generally associated with a lower degree of iron overload compared with beta-thalassemia. As a result, patients with alpha-thalassemia may exhibit a relatively more favorable response to deferasirox therapy [26].

Deferasirox was generally well tolerated with a manageable safety profile throughout the study. The most common side effect associated with DFX is nausea, which was reported in 9.52% of our patients. Comparatively, in other studies, the incidence of nausea was reported to be around 7.20% [12]. Additionally, skin rash was found to be another common adverse event, occurring in approximately 8.00% of cases in other studies [12]. although it was not observed in our study. Our study revealed that patients experienced increased alanine aminotransferase (ALT) and increased creatinine levels, approximately 4.76%, and 16.67%, respectively. Importantly, these abnormalities were resolved without the necessity of discontinuing treatment. This suggests that while these adverse events may occur in a minority of patients, they can often be managed effectively without interrupting DFX therapy. Nonetheless, regular monitoring for changes in liver enzymes and kidney function remains crucial during treatment with DFX.

The strength of our study lies in its utilization of real-world data derived from our database, providing insights into the long-term outcomes of DFX monotherapy as a second-line treatment in patients with TDT which is an effective and manageable adverse effect. However, it is important to acknowledge the limitations of our study. One notable limitation is its retrospective nature, which may have led to incomplete documentation of non-serious adverse events in the database. This could potentially introduce bias or affect the comprehensiveness of the adverse event profile reported in our study. Due to health care resource constraints, patients have not received MRI scans for evaluation of LIC and cardiac T2*. Incorporating these modalities into future national programs may help optimize treatment outcomes.

Based on our findings, we suggest the following clinical recommendations. First, switching to deferasirox (DFX) should be considered in patients with an inadequate response to first-line iron chelation therapy, defined as a serum ferritin level greater than 2500 ng/mL after one year of prior iron chelator use, or a decrease in serum ferritin of less than 15% after two years of treatment. DFX should be initiated at a starting dose of 20 mg/kg/day, with gradual dose escalation of 5–10 mg/kg every three to six months according to iron stores. A dose of approximately 30 mg/kg/day is typically required to achieve a negative iron balance. Following the switch, careful monitoring of serum ferritin, and relevant safety parameters is recommended to optimize treatment outcomes and minimize adverse effects. Moreover, attention to adherence and patient education, are essential for maximizing the efficacy of DFX therapy [21].

## 5. Conclusions

Our study reaffirmed that deferasirox (DFX) is an effective oral iron chelator in patients with thalassemia, demonstrating acceptable adverse events. Additionally, we observed that being an adult (age > 15 years) and having initially lower serum ferritin (SF) levels were associated with a better response to DFX treatment. These factors can potentially aid clinicians in identifying patients who are more likely to benefit from DFX therapy, thereby optimizing treatment outcomes in thalassemia patients. Additionally, prospective long-term studies incorporating advanced iron monitoring are warranted, which may inform future guideline updates to reflect the real-world efficacy of DFX.

## Figures and Tables

**Table 1:** Baseline Characteristics.

Characteristic	All Patients (*n* = 42)
Age, median (IQR)	35.5 (13–57)
Gender, *n* (%)	
Male	22 (52.38%)
Female	20 (47.62%)
Underlying disease, *n* (%)	
No other underlying disease	29 (69.05%)
DM	1 (2.38%)
HTN	2 (4.76%)
DLP	1 (2.38%)
Thalassemia type, *n* (%)	
Alpha thalassemia	11 (26.19%)
Hb H disease	10 (90.91%)
Hb H/CS	1 (9.09)
Beta thalassemia	31 (73.81%)
Beta thalassemia/Hb E	19 (61.29%)
Beta thalassemia major	12 (38.71%)
Splenectomy, *n* (%)	12 (28.57%)
Number of transfusions, median (IQR), unit/month	0.75 (0.5–1)
Previous iron chelator, *n* (%)	
Deferoxamine monotherapy	14 (33.33%)
Deferiprone monotherapy	27 (64.29%)
Deferoxamine + Deferiprone	1 (2.38%)
Baseline SF, median (range), ng/mL	2516 (1712–3065)
Pretransfusion Hb, mean ± SD, g/dL	8.130 ± 1.600
Maximum dose of DFX, median (range), mg/kg/day	27.19 (21.73–32.37)

Abbreviations: DM, Diabetes mellitus; HTN, Hypertension; DLP, Dyslipidemia; SF, serum ferritin; DFX, Deferasirox; IQR, interquartile range.

**Table 2:** Associated Factors of Ferritin Response Group from Univariate and Multivariate Analyses.

Parameters	Total (*n* = 42)	Ferritin Response Group [*n*, (%)]	Univariate			Multivariate		
			OR	95% CI	*p* Value	OR	95% CI	*p* Value
Gender								
Male	22	11 (50.00%)	1	0.29–3.41	1.000			
Female	20	10 (50.00%)	Reference					
Age (years)								
Adult	30	19 (63.33%)	8.64	1.56–47.78	0.013	7.13	1.05–48.49	0.045
Pediatrics	12	2 (16.67%)	Reference					
Thalassemia type								
Alpha thalassemia	11	9 (81.82%)	7.12	1.28–39.58	0.025	4.46	0.79–25.27	0.091
Beta thalassemia	31	12 (38.71%)	Reference					
Splenectomy								
Intact spleen	30	16 (53.33%)	1.6	0.41–6.29	0.501			
Splenectomized	12	5 (41.67%)	Reference					
Reasons for switching to DFX								
Cannot response target SF	29	14 (48.28%)	1.25	0.33–4.63	0.738			
Exhibiting adverse events	13	7 (53.85%)	Reference					
Initial serum ferritin (ng/mL)			0.99	0.99–1.00	0.083	0.93	0.87–1.00	0.039
Starting dose of DFX								
<20 mg/kg/day	19	12 (63.16%)	2.66	0.75–9.48	0.130			
≥20 mg/kg/day	23	9 (39.13%)	Reference					
Pre-transfusion Hb								
<7 g/dL	10	4 (40.00%)	Reference					
≥7 g/dL	32	17 (53.12%)	1.70	0.39–7.32	0.476			

Abbreviations: SF, serum ferritin; DFX, Deferasirox; OR, odds ratio; CI, confidence interval.

## Data Availability

The original contributions presented in this study are included in the article. Further inquiries can be directed to the corresponding author(s).

## Abbreviations

DFX	deferasirox
SF	serum ferritin
DM	Diabetes mellitus
HTN	Hypertension
DLP	Dyslipidemia
eGFR	estimated glomerular filtration rate
HPLC	high-performance liquid chromatography
TDT	transfusion-dependent thalassemia
NLEM	National List of Essential Medicines
TIF	Thalassemia International Federation
DFP	Deferiprone
DFO	Deferoxamine
IQR	interquartile range
LIC	liver iron concentration
OR	odds ratio
CI	confidence interval
ALT	alanine aminotransferase
Hb	Hemoglobin
